# Left bundle branch pacing preserved left ventricular myocardial work in patients with bradycardia

**DOI:** 10.3389/fcvm.2023.1201841

**Published:** 2023-09-14

**Authors:** Huang-Chung Chen, Wen-Hao Liu, Yung-Lung Chen, Wei-Chieh Lee, Yen-Nan Fang, Shaur-Zheng Chong, Mien-Cheng Chen

**Affiliations:** ^1^ Division of Cardiology, Department of Internal Medicine, Kaohsiung Chang Gung Memorial Hospital, College of Medicine, Chang Gung University, Kaohsiung, Taiwan; ^2^ Division of Cardiovascular Medicine, Chi-Mei Medical Center, Tainan, Taiwan

**Keywords:** global longitudinal strain, left bundle branch pacing, mechanical synchrony, myocardial work efficiency, his bundle pacing

## Abstract

**Background:**

Left bundle branch pacing (LBBP) is an emerging physiological pacing modality. Left ventricular (LV) myocardial work (MW) incorporates afterload and LV global longitudinal strain to estimate global and segmental myocardial contractility. However, the effect of LBBP on LV MW remains unknown. This study aimed to evaluate the impact of LBBP on LV MW in patients receiving pacemaker for bradyarrhythmia.

**Methods:**

We prospectively enrolled 70 bradycardia patients with normal LV systolic function receiving LBBP (*n* = 46) and non-selective His-bundle pacing (NS-HBP) (*n* = 24). For comparative analysis, patients receiving right ventricular pacing (RVP) (*n* = 16) and control subjects (*n* = 10) were enrolled. Two-dimensional speckle tracking echocardiography was performed. The LV pressure-strain loop was non-invasively constructed to assess global LV MW.

**Results:**

After 6-month follow-up, LBBP group (with >40% ventricular pacing during 6 months) had shorter peak strain dispersion (PSD) compared with RVP group, and higher LV global longitudinal strain compared with RVP group and NS-HBP group, but had no difference in left intraventricular mechanical dyssynchrony, including septal-to-posterior wall motion delay and PSD, compared with NS-HBP group. During ventricular pacing, LBBP group had higher global MW index (GWI) (2,189 ± 527 vs. 1,493 ± 799 mmHg%, *P* = 0.002), higher global constructive work (GCW) (2,921 ± 771 vs. 2,203 ± 866 mmHg%, *P* = 0.009), lower global wasted work (GWW) (211 ± 161 vs. 484 ± 281 mmHg%, *P* < 0.001) and higher global MW efficiency (GWE) (91.4 ± 5.0 vs. 80.9 ± 8.3%, *P* < 0.001) compared with RVP group, and had lower GWW (211 ± 161 vs. 406 ± 234 mmHg%, *P* < 0.001) and higher GWE (91.4 ± 5.0 vs. 86.4 ± 8.1%, *P* < 0.001) compared with NS-HBP group.

**Conclusions:**

In this study we found that in patients with mid-term (6-month) high ventricular pacing burden (>40%), LBBP preserved more LV MW compared with NS-HBP and RVP. Further studies are warranted to assess the association between LV MW and long-term clinical outcomes in LBBP with high ventricular pacing burden.

## 1. Introduction

Permanent pacemaker is an effective therapy for patients with symptomatic bradyarrhythmia, including sinus nodal dysfunction and atrioventricular block. Right ventricular pacing (RVP) over the right ventricular (RV) apex or the RV septum has been reported to increase the risks of atrial fibrillation, heart failure (HF) hospitalization, or mortality (1, 2). Previous studies have demonstrated that acute RVP leads to electrical and mechanical dyssynchrony of left ventricle (LV) (3, 4), and long-term pacing also results in histopathological changes and LV remodeling (5, 6). Recently, conduction system pacing, such as His-bundle pacing (HBP) and left bundle branch pacing (LBBP), are emerging physiological pacing strategies (7, 8). In clinical practice, LBBP is more feasible than HBP, owing to more stable threshold during follow-up (8). Theoretically, LBBP could directly stimulate left bundle branch area to synchronize LV contraction in patients with left bundle branch block. However, myocardial deformation imaging studies with LV global longitudinal strain (GLS) for intraventricular or interventricular synchrony for LBBP compared with HBP and RVP are limited (9–12). Moreover, LV myocardial work (MW), derived from non-invasive LV pressure-strain loop estimations that incorporates both information on afterload and LV GLS, can overcome the limitations and shortcomings of load dependency of speckle tracking echocardiography (STE) and may provide additional information regarding dyssynchronous contraction, segmental work, and myocardial contractility (13, 14). Furthermore, global LV MW efficiency has been reported to be associated with long-term survival in patients after ST-segment elevation myocardial infarction (15). However, study to compare the difference in the impact on the LV MW among LBBP, RVP and HBP is lacking (13, 14). Accordingly, we conducted this prospective study to evaluate the immediate and mid-term effects of LBBP on the intraventricular and interventricular mechanical synchronies, and LV MW compared with HBP and RVP in patients receiving transvenous permanent pacemaker implantation for symptomatic bradyarrhythmia.

## 2. Materials and methods

### 2.1 Study subjects

Patients with symptomatic sinus nodal dysfunction and atrioventricular block receiving conduction system pacing were prospectively enrolled in our hospital between July, 2020 and December, 2021. For comparative analysis of case-control study, gender- and comorbidity-matched patients receiving transvenous RVP for symptomatic bradyarrhythmia and gender- and comorbidity-matched control subjects without pacemaker between July, 2020 and December, 2021 were also enrolled in this study. The exclusion criteria included patients with: (1) any history of HF with left ventricular ejection fraction (LVEF) <50% due to severe valvular heart disease or cardiomyopathy, (2) permanent atrial fibrillation, (3) left bundle branch block, (4) end-stage renal disease, and (5) malignancy with expected lifespan <1 year.

### 2.2 Procedures of conduction system pacing

The detailed procedures are provided in Supplementary Methods: Procedures of conduction system pacing.

### 2.3 Echocardiographic data acquisition

Echocardiography was performed within 7 days and at 6-month follow-up after the pacemaker implantation. All echocardiographic measurements were obtained initially during sinus or intrinsic rhythm for patients with normal atrioventricular conduction at the time of echocardiography, and then obtained during the DDD pacing mode with an atrioventricular delay of 100–120 ms to ensure complete capture by pacing without fusion in patients with normal atrioventricular conduction, at 2-fold output of pacing threshold, and a pacing rate between 80 and 110 beats/min. Because of inconsistent pacing threshold of HBP group during follow-up, echocardiographic images were only acquired during non-selective HBP (NS-HBP). Transthoracic echocardiographic images were recorded using a Vivid E9 ultrasound system (GE Healthcare, Milwaukee, WI, US) with M5S 3.5 MHz transducers. All echocardiographic measurements were obtained for at least 3 consecutive beats for sinus and pacing rhythm, and 5 consecutive beats for atrial fibrillation rhythm, and were stored with digital loops for offline analysis with EchoPAC (Version 202, GE Healthcare, Chicago, IL, US).

Based on the recommendations for cardiac chamber quantification by echocardiography (16), left atrial diameters, and LV dimensions and volumes were measured using two-dimensional echocardiography, and LVEF was calculated by biplane Simpson's method from the apical 4-chamber and 2-chamber views. The mean E/e' ratio (the average value of septal E/e' and lateral E/e'), tricuspid annular plane systolic excursion (TAPSE), and S' wave of the RV free wall (FW) were also obtained.

### 2.4 Evaluations of intraventricular and interventricular synchronies

The intraventricular and interventricular synchronies were evaluated by pulsed-wave Doppler imaging echocardiography, tissue Doppler (Ts) imaging and STE. The regional durations of time measured for the basal segments in the LVFW, RVFW and interventricular septum (IVS) were from the start of QRS to the peak velocity of S' wave of Ts in the LVFW (Ts-LVFW), RVFW (Ts-RVFW) and septum (Ts-IVS), respectively (17).

**Figure 1:**
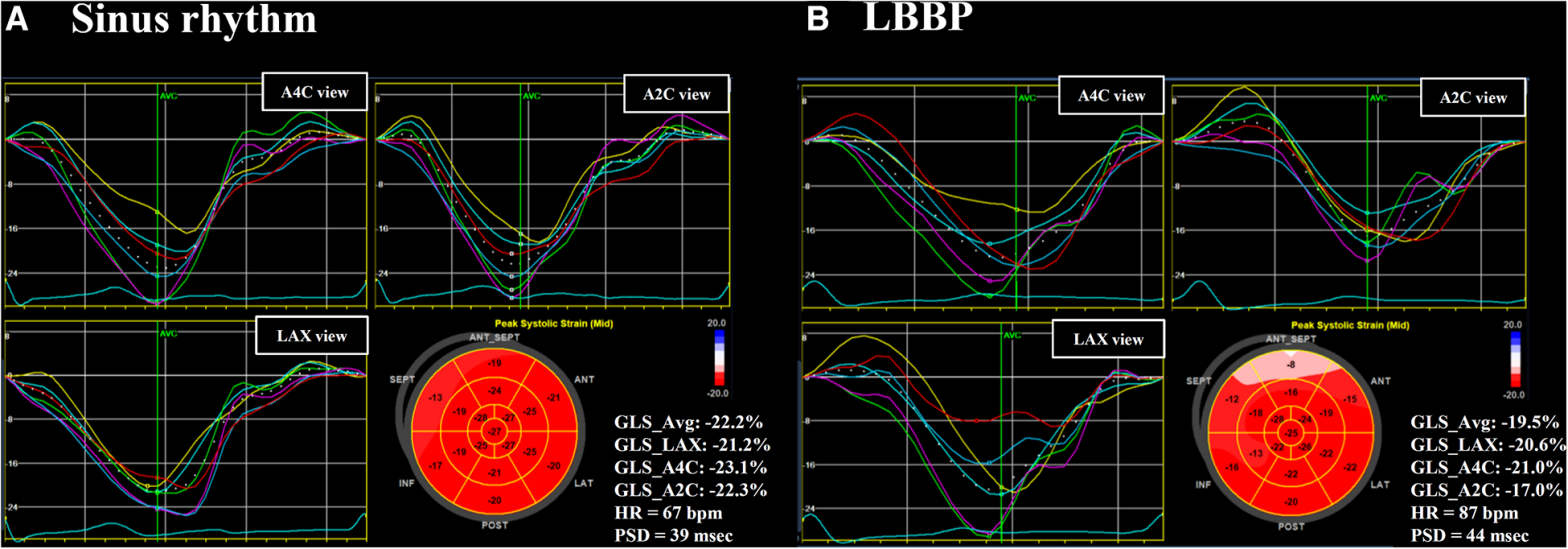
Left ventricular GLS and PSD. Left ventricular (LV) GLS and PSD assessed in a patient during sinus rhythm (**A**) and during LBBP (**B**). The value of GLS in apical three-chamber (LAX) view, apical four-chamber (A4C) view, and apical two-chamber (A2C) view were calculated individually. A bull's-eye plot was created of peak LV GLS for each myocardial segment. GLS, global longitudinal strain; HR, heart rate; LBBP, left bundle branch pacing; PSD, peak strain dispersion.

The LV GLS, peak strain dispersion (PSD), septal-to-posterior wall motion delay (SPWMD), and difference of Ts between LVFW and IVS (Ts-LVFW-IVS) were used as LV mechanical synchrony parameters (3, 4, 9, 10, 17, 18). The value of Ts-LVFW-IVS means the time of S' in the LVFW minus the time of S' in the septum. The LV GLS measured by STE at a frame rate of 50–80 frames/s, and the apical four-chamber (A4C) view, apical three-chamber (LAX) view, and apical two-chamber (A2C) view of STE were recorded as well as electrocardiography. Peak LV GLS was then performed using echocardiography software that divided the myocardium into 6 segments in each view, creating graphs of shortening over the cardiac cycle. A bull's-eye plot was created of peak LV GLS for each myocardial segment (Figure 1). That absolute values of GLS were used when comparison among different study groups. PSD is defined as the standard deviation of time to peak longitudinal systolic strain of LV segments (Figure 1). The difference of Ts between Ts-RVFW and Ts-IVS (Ts-RVFW-IVS) was used as RV intraventricular mechanical synchrony parameter (10). For interventricular synchrony, the value of LV pre-ejection period (LV PEP), interventricular mechanical delay (IVMD), and different of Ts between LVFW and RVFW (Ts-LVFW-RVFW) were calculated (10, 17). Pulsed-wave Doppler flow velocity signal was recorded from the LV outflow tract, and LV PEP were measured as the time intervals between the Q-wave on the surface ECG and the onset of Doppler flow. IVMD was defined as the time difference between LV PEP and RV PEP, which were the periods from the start of QRS wave to the start of pulsed-wave Doppler flow of LV and RV outflow tracts, respectively. The values of LV PEP >140 ms and IVMD >40 ms were both considered indicative of interventricular dyssynchrony (17, 19).

### 2.5 Quantification of LV myocardial work

**Figure 2:**
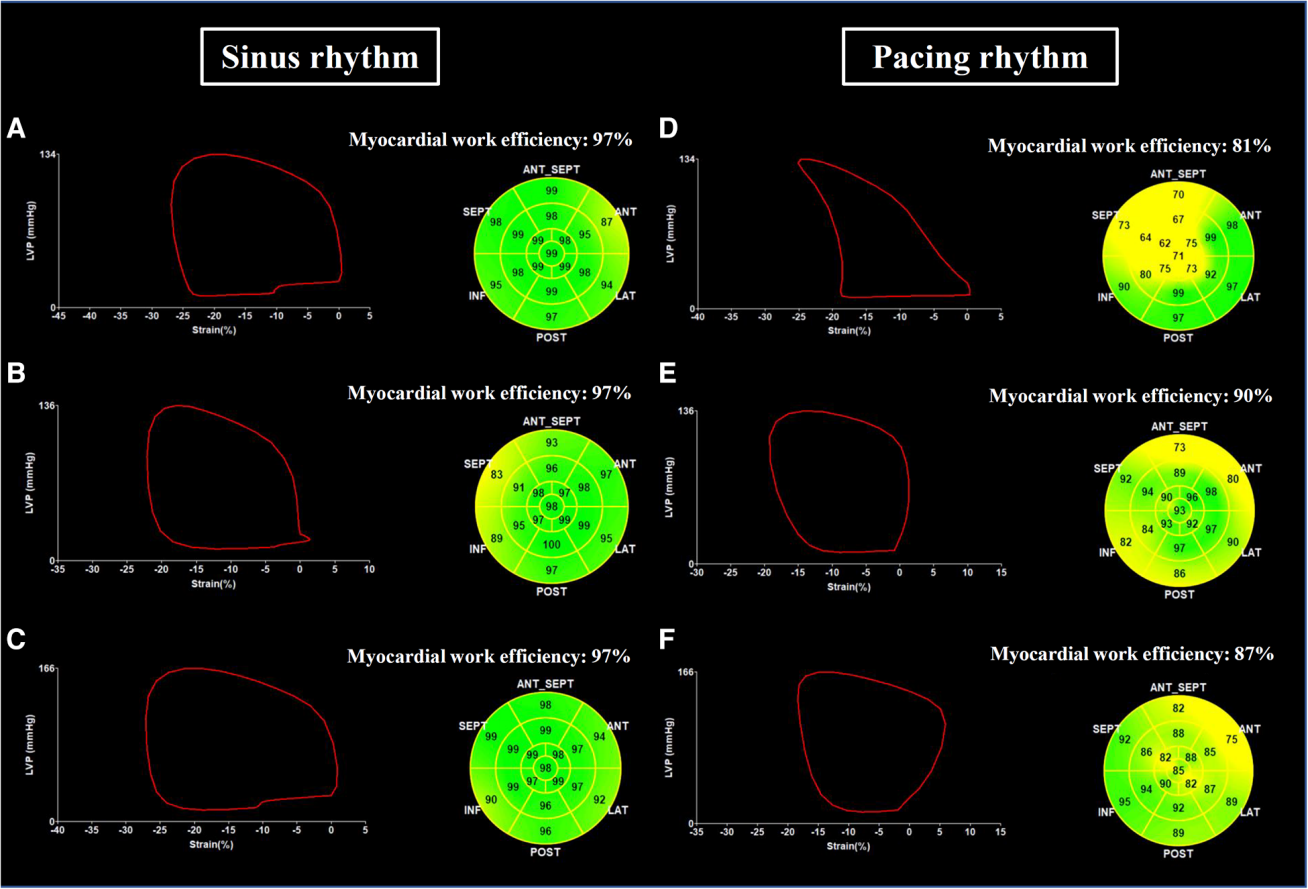
Examples of LV pressure-strain loop and myocardial work efficiency. Global left ventricular pressure-strain loop and myocardial work efficiency assessed in a patient with RVP during sinus rhythm (**A**) and during ventricular pacing (**D**), in a patient with LBBP during sinus rhythm (**B**) and during ventricular pacing (**E**), and in a patient with NS-HBP during sinus rhythm (**C**) and during ventricular pacing (**F**) NS-HBP, non-selective His-bundle pacing; LBBP, left bundle branch pacing; RVP, right ventricular pacing.

Global LV MW data were quantified using non-invasive LV pressure-strain loop estimations and the area within the LV pressure–strain loop represents MW (13, 14). Non-invasive blood pressure recordings to estimate LV pressure were measured by a sphygmomanometer to obtain the brachial cuff systolic blood pressure immediately prior to the echocardiographic study (14). The quantification of MW was conducted by EchoPAC (Version 202, GE Healthcare, Chicago, IL, US) according to the following steps. First, the duration of isovolumic and ejection phases was defined by valvular timing (the opening and closing time of mitral and aortic valves) according to pulse-wave Doppler imaging. Then, the LV pressure-strain loop was constructed automatically with the combination of LV strain and non-invasive blood pressure measurements adjusted by the duration of the isovolumic and ejection phases (Figure 2) (14). Global myocardial work index (GWI) was calculated as the average of MW from the three apical views using GLS pressure loop from mitral valve closure to mitral valve opening. Global constructive work (GCW) was defined as the MW performed by LV segmental shortening during systole or by LV segmental lengthening during isovolumic relaxation. Global wasted work (GWW) was defined as the MW performed by LV segmental lengthening during systole or MW performed by LV segmental shortening during isovolumic relaxation. Global myocardial work efficiency (GWE) was defined as the ratio of GCW to total work (GCW plus GWW), expressed as a percentage (Figure 2).

### 2.6 Intra- and inter-observer variability

Intra- and inter-observer variabilities were calculated by intraclass correlation coefficient and the standard error of measurement. For intra-observer analysis of the LV synchrony data, 18 random measurements were reanalyzed, with a period of 2–4 weeks between the first and second analyses. Intraclass correlation coefficients for intra-observer agreement were 0.933 (*P* < 0.001) for GLS and 0.948 (*P* < 0.001) for GWI. Inter-observer variability was performed by a different cardiologist repeating measurements from the same images. For interobserver analysis of the LV synchrony data, 18 random measurements were analyzed. The intraclass correlation coefficients for interobserver agreement were 0.986 (*P* < 0.001) for GLS and 0.910 (*P* < 0.001) for GWI.

### 2.7 Statistical analysis

Continuous variables are expressed as mean ± standard deviation, and categorical variables were expressed as numbers and percentages. Continuous variables were compared using unpaired Student's *t*-test or the Kruskal–Wallis test in case of a non-normal distribution. Categorical variables were compared using Chi-square test or Fisher exact test as appropriate. Paired sample *t*-test or Wilcoxon test in case of a non-normal distribution was performed to assess differences of paired samples. Comparison of the continuous variables among groups was performed by one-way analysis of variance (ANOVA), with Bonferroni correction when significant events were detected. A two-sided *P*-value <0.05 was considered statistically significant. All statistical analyses were performed using the SPSS software (version 22.0; SPSS Inc., Chicago, IL, USA).

## 3. Result

### 3.1 Study enrollment

This study recruited 109 consecutive patients receiving transvenous permanent conduction system pacing. Thirty-nine patients were excluded, including 6 patients with history of HF due to severe valvular heart disease or cardiomyopathy, 7 patients with end-stage renal disease, 6 patients with permanent atrial fibrillation, 3 patients that refused to join the study and 17 patients with left ventricular septal pacing (LVSP) according to the reported criteria (20–22). Finally, 70 patients with physiological pacing, including 24 patients with NS-HBP and 46 patients with LBBP, were enrolled. For comparative analysis of case-control study, 16 patients with RVP and 10 subjects without pacemaker (control group) were also enrolled.

### 3.2 Patient characteristics

Table 1 lists the clinical characteristics of the study patients. The mean age of the entire study subjects was 74 ± 9 years and 51.0% of the study subjects were male. The NS-HBP group was older than the LBBP and control groups (Table 1). The NS-HBP and RVP groups had a higher prevalence of atrial fibrillation, and a higher prescription rate of antiarrhythmic and anticoagulants compared with the control and LBBP groups (Table 1). The control group had a higher prescription rate of angiotensin converting enzyme inhibitors/angiotensin receptor blockers compared with the other 3 groups (Table 1).

**Table 1:** Baseline characteristics and procedural parameters of the study subjects.

Group	Control (*n* = 10)	RVP (*n* = 16)	LBBP (*n* = 46)	NS-HBP (*n* = 24)	*P*-value
Age, (years)	66 ± 8	76 ± 5	73 ± 11	79 ± 7^†^^§^	0.002
Male	6 (60)	5 (31.3)	28 (60.9)	10 (41.7)	0.142
Body mass index, (kg/m^2^)	27 ± 4	24 ± 4	26 ± 4	26 ± 4	0.376
Comorbidities					
Hypertension	7 (70.0)	13 (81.3)	34 (73.9)	21 (87.5)	0.539
Diabetes mellitus	0 (0)	5 (31.3)	18 (39.1)	11 (45.8)	0.074
Coronary artery disease	1 (10.0)	2 (12.5)	8 (17.4)	0 (0)	0.196
Heart failure	0 (0)	2 (12.5)	8 (17.4)	5 (20.8)	0.467
Paroxysmal atrial fibrillation	1 (10.0)	10 (62.5)	10 (21.7)	14 (58.3)	0.001
Ischemic stroke	0 (0)	3 (18.3)	1 (2.2)	2 (8.3)	0.088
Chronic kidney disease^a^	4 (40.0)	9 (56.3)	18 (39.1)	13 (54.2)	0.505
Medications					
Beta-blocker	6 (60.0)	10 (62.5)	18 (39.1)	8 (33.3)	0.190
ACEi/ARB	8 (80.0)	7 (43.8)	15 (32.6)	12 (50.0)	0.047
Antiarrhythmic drug	2 (20.0)	10 (62.5)	8 (17.4)	10 (41.7)	0.005
Amiodarone/Dronedarone	1 (10)	7 (43.8)	7 (15.2)	9 (37.5)	0.037
Anticoagulants	1 (10.0)	12 (75.0)	8 (17.4)	14 (58.3)	<0.001
Laboratory data					
Serum creatinine, (mg/dl)	1.0 ± 0.2	1.3 ± 0.6	1.5 ± 1.7	1.3 ± 0.8	0.756
eGFR, (ml/min/1.73 m^2^)	69 ± 18	53 ± 22	65 ± 30	62 ± 29	0.442
Procedural and pacing parameters					
Patients with AVB	0 (0)	0 (0)	31 (67.4)	3 (12.5)	<0.001
Pre-existing RBBB	0 (0)	1 (6.3)	15 (32.6)	3 (12.5)	<0.001
Intrinsic QRS duration, (ms)	96 ± 6	89 ± 12	107 ± 33	96 ± 24	0.075
Pacing QRS duration, (ms)	N/A	145 ± 14^§^^※^	114 ± 11	116 ± 12	<0.001
Ventricular pacing during follow-up, (%)	N/A	17 ± 35	59 ± 47^‡^^※^	14 ± 29	<0.001

Data are presented as mean ± SD or number (%) of patients.

ACEi/ARB, angiotensin converting enzyme inhibitors/angiotensin receptor blocker; AVB, atrioventricular block; eGFR, estimated glomerular filtration rate; LBBP, left bundle branch pacing; N/A, not applicable; NS-HBP, non-selective His-bundle pacing; RBBB, right bundle branch block; RVP, right ventricular pacing.

^a^ Defined as eGFR lower than 60 ml/min/1.73 m^2^ without renal replacement therapy.

^†^ *P* < 0.05 when compared with control subjects.

^‡^ *P* < 0.05 when compared with RVP.

^§^ *P* < 0.05 when compared with LBBP.

^※^ *P* < 0.05 when compared with NS-HBP.

### 3.3 Procedural and pacing parameters

**Figure 3:**
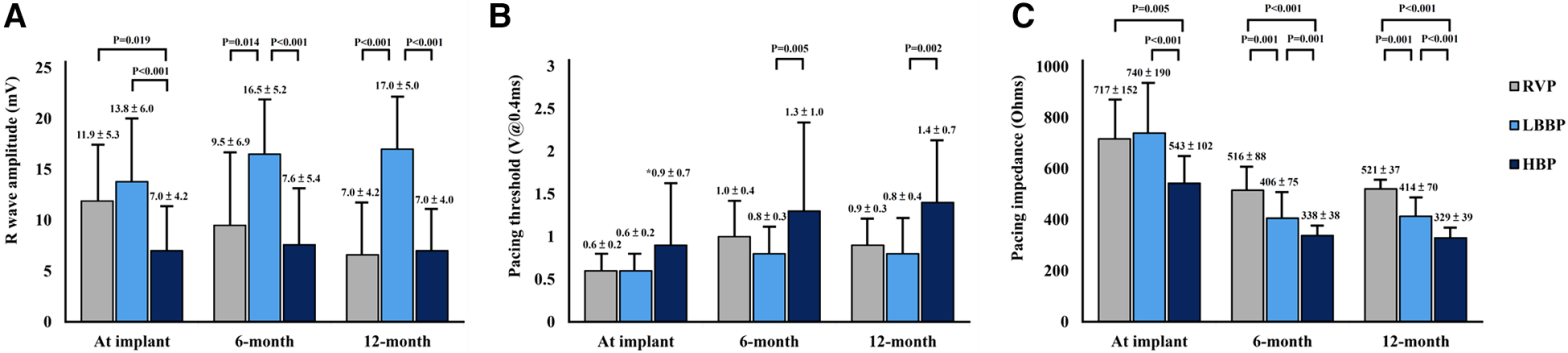
Pacing parameters of pacemakers. Comparison of pacing parameters including R-wave amplitude (**A**), pacing threshold (**B**), and pacing impedance (**C**) among RVP, LBBP and NS-HBP groups at implant, 6-month and 12-month follow-ups. Abbreviations as in Figure 2.

The LBBP group had a higher prevalence of atrioventricular block and pre-existing RBBB, and a higher burden of ventricular pacing during follow-up compared with the other 3 groups (Table 1). In the LBBP group, the stimulus to left ventricular activation time was 69 ± 8 ms, and the LBB potential to QRS was 23 ± 6 ms. The pacing QRS duration was wider in the RVP group compared with the LBBP and NS-HBP groups (145 ± 14 vs. 114 ± 11 vs. 116 ± 12 ms, respectively, *P* < 0.001) (Table 1). About pacing parameters, LBBP group had higher R wave amplitude at implant and follow-up, lower pacing threshold at follow-up, and higher pacing impedance at implant and follow-up compared with NS-HBP group (Figure 3).

### 3.4 Echocardiographic parameters and cardiac functions during sinus or intrinsic rhythm and atrial synchronized ventricular pacing

Conventional echocardiographic findings at implant and at 6-month follow-up are summarized in Table 2. The left atrial size significantly increased during ventricular pacing at 6-month follow-up in the RVP group compared with the LBBP and NS-HBP groups (46 ± 15 vs. 39 ± 8 vs. 38 ± 8 mm, *P* = 0.031) (Table 2). The LV end-diastolic and end-systolic volumes during ventricular pacing did not differ among the RVP, LBBP and NS-HBP groups at implant and at 6-month follow-up. Likewise, LVEF during ventricular pacing did not differ among the RVP, LBBP and NS-HBP groups at implant and at 6-month follow-up. The mean E/e', TAPSE, and S' wave of RVFW during ventricular pacing also did not differ among the RVP, LBBP and NS-HBP groups at implant and at 6-month follow-up (Table 2).

**Table 2:** Echocardiographic parameters, cardiac function and intraventricular and interventricular mechanical synchronies during sinus or intrinsic and synchronized ventricular pacing in the control and different pacing groups at implant and 6-month follow-up.

	At implant					6-month follow-up				
	Control	RVP	LBBP	NS-HBP	*P*-value	Control	RVP	LBBP	NS-HBP	*P*-value
Chamber size										
LA size (sinus or intrinsic rhythm), (mm)	39 ± 4	38 ± 8	37 ± 5	39 ± 5	0.570	41 ± 10	41 ± 7	37 ± 5	39 ± 6	0.311
LA size (ventricular pacing), (mm)	N/A	36 ± 5	36 ± 5	39 ± 7	0.174	N/A	46 ± 15^※^	39 ± 8	38 ± 8	0.031
LVEDV (sinus or intrinsic rhythm), (ml)	122 ± 21	99 ± 27	101 ± 31	108 ± 39	0.267	103 ± 17	96 ± 16	111 ± 38	115 ± 26	0.268
LVEDV (ventricular pacing), (ml)	N/A	98 ± 12	99 ± 30	112 ± 31	0.202	N/A	99 ± 37	106 ± 37	116 ± 41	0.395
LVESV (sinus or intrinsic rhythm), (ml)	35 ± 10	31 ± 9	33 ± 14	34 ± 14	0.806	30 ± 6	30 ± 6	36 ± 18	37 ± 9	0.261
LVESV (ventricular pacing), (ml)	N/A	30 ± 8	33 ± 15	35 ± 17	0.680	N/A	33 ± 32	36 ± 20	40 ± 16	0.598
LV function and synchrony										
LVEF (Biplane) (sinus or intrinsic rhythm), (%)	66 ± 6	65 ± 7	65 ± 7	64 ± 8	0.934	66 ± 6	68 ± 4	67 ± 7	66 ± 9	0.887
LVEF (Biplane) (ventricular pacing), (%)	N/A	62 ± 11	62 ± 8	62 ± 7	0.984	N/A	62 ± 13	63 ± 8	62 ± 9	0.787
Mean E/e’ (sinus or intrinsic rhythm)	9.9 ± 3.5	11.1 ± 3.8	12.1 ± 7.1	11.1 ± 3.7	0.686	8.4 ± 1.2	11.6 ± 4.2	11.9 ± 5.6	11.9 ± 4.2	0.198
Mean E/e’ (ventricular pacing)	N/A	12.1 ± 4.8	11.6 ± 6.6	10.2 ± 3.8	0.531	N/A	11.0 ± 4.2	12.7 ± 5.3	12.3 ± 4.8	0.513
SPWMD (sinus or intrinsic rhythm), (ms)	66 ± 16	84 ± 26	67 ± 20	79 ± 33	0.160	66 ± 21	67 ± 21	61 ± 26	73 ± 23	0.456
SPWMD (ventricular pacing), (ms)	N/A	122 ± 39^§^^※^	78 ± 22	82 ± 15	<0.001	N/A	113 ± 35^§^^※^	72 ± 23	86 ± 24	<0.001
PSD (sinus or intrinsic rhythm), (ms)	57 ± 20	63 ± 16	56 ± 13	55 ± 21	0.545	50 ± 13	48 ± 24	50 ± 17	57 ± 25	0.577
PSD (ventricular pacing), (ms)	N/A	63 ± 17^※^	53 ± 15	47 ± 20	0.027	N/A	65 ± 26^§^^※^	48 ± 14	42 ± 22	0.002
GLS (sinus or intrinsic rhythm), (-%)	20.7 ± 3.2	19.6 ± 2.9	19.4 ± 2.7	20.2 ± 5.4	0.748	21.6 ± 4.2	22.3 ± 2.5	21.1 ± 2.3	21.4 ± 3.9	0.721
GLS (ventricular pacing), (-%)	N/A	17.5 ± 2.3	18.4 ± 3.2	17.3 ± 4.2	0.400	N/A	16.6 ± 5.4	20.0 ± 2.8^‡^	18.5 ± 5.8	0.033
GLS LAX (sinus or intrinsic rhythm), (-%)	20.5 ± 4.3	19.0 ± 2.9	19.1 ± 3.3	19.6 ± 5.6	0.770	20.1 ± 4.7	19.3 ± 3.6	20.5 ± 3.7	20.6 ± 4.0	0.771
GLS LAX (ventricular pacing), (-%)	N/A	17.4 ± 2.5	17.8 ± 4.1	16.7 ± 4.3	0.557	N/A	15.7 ± 5.2	19.7 ± 3.5^‡^	17.7 ± 5.8	0.015
GLS A4C (sinus or intrinsic rhythm), (-%)	21.5 ± 3.3	20.0 ± 3.8	19.6 ± 2.6	20.6 ± 5.6	0.585	21.8 ± 4.5	22.2 ± 4.9	21.2 ± 3.0	22.3 ± 3.6	0.764
GLS A4C (ventricular pacing), (-%)	N/A	17.0 ± 3.2	18.9 ± 3.4	18.4 ± 4.5	0.226	N/A	16.3 ± 5.2	20.4 ± 3.4^‡^	18.8 ± 7.2	0.028
GLS A2C (sinus or intrinsic rhythm), (-%)	20.2 ± 3.7	19.9 ± 3.9	19.5 ± 4.0	20.3 ± 6.5	0.944	23.0 ± 5.5	23.8 ± 3.2	21.5 ± 3.6	21.5 ± 5.6	0.350
GLS A2C (ventricular pacing), (-%)	N/A	18.2 ± 3.4	18.6 ± 3.9	16.9 ± 5.6	0.330	N/A	18.4 ± 6.1	20.1 ± 3.7	18.0 ± 6.7	0.271
Ts-LVFW-IVS (sinus or intrinsic rhythm), (ms)	35 ± 53	47 ± 48	20 ± 38	39 ± 42	0.201	31 ± 32	36 ± 29	23 ± 33	31 ± 35	0.587
Ts-LVFW-IVS (ventricular pacing), (ms)	N/A	83 ± 55^§^^※^	16 ± 37	29 ± 39	<0.001	N/A	45 ± 50^§^^※^	18 ± 38	15 ± 30	0.042
RV function and synchrony										
TAPSE (sinus or intrinsic rhythm), (mm)	25 ± 4	24 ± 4	22 ± 5	23 ± 4	0.223	26 ± 3	25 ± 4	24 ± 4	23 ± 5	0.162
TAPSE (ventricular pacing), (mm)	N/A	23 ± 4	23 ± 4	27 ± 18	0.237	N/A	21 ± 4	22 ± 3	23 ± 4	0.180
S’ wave of RVFW (sinus or intrinsic rhythm), (cm/s)	13.6 ± 2.2	12.1 ± 2.0	12.3 ± 2.6	13.0 ± 3.0	0.420	12.7 ± 1.5	11.3 ± 2.8	11.0 ± 2.2	11.8 ± 2.3	0.241
S’ wave of RVFW (ventricular pacing), (cm/s)	N/A	12.3 ± 2.3	12.9 ± 2.5	13.6 ± 3.8	0.382	N/A	10.7 ± 2.9	11.7 ± 2.6	11.9 ± 2.4	0.375
Ts-RVFW-IVS (sinus or intrinsic rhythm), (ms)	8 ± 24	−3 ± 29	8 ± 48	21 ± 28	0.330	5 ± 20	10 ± 31	13 ± 32	8 ± 33	0.887
Ts-RVFW-IVS (ventricular pacing), (ms)	N/A	15 ± 20	17 ± 22	14 ± 16	0.807	N/A	17 ± 41	15 ± 26	7 ± 33	0.548
Interventricular synchrony										
LV PEP (sinus or intrinsic rhythm), (ms)	72 ± 17	83 ± 10	103 ± 24	91 ± 11	0.057	84 ± 27	88 ± 5	94 ± 11	100 ± 21	0.326
LV PEP (ventricular pacing), (ms)	N/A	114 ± 30	111 ± 19	118 ± 23	0.921	N/A	141 ± 16	104 ± 23^‡^	119 ± 24	0.011
IVMD (sinus or intrinsic rhythm), (ms)	−3	−10 ± 13	−3 ± 10	−4 ± 9	0.886	−8 ± 7	−1 ± 22	−10 ± 19	5 ± 8	0.344
IVMD (ventricular pacing), (ms)	N/A	14 ± 13	−6 ± 6^‡^	4 ± 1	0.027	N/A	21 ± 24	−8 ± 11^‡^	8 ± 11	<0.001
Ts-LVFW-RVFW (sinus or intrinsic rhythm), (ms)	18 ± 50	51 ± 52	9 ± 55	22 ± 54	0.121	25 ± 44	27 ± 52	10 ± 46	23 ± 53	0.648
Ts-LVFW-RVFW (ventricular pacing), (ms)	N/A	58 ± 60	1 ± 46^‡^	16 ± 38	0.002	N/A	28 ± 45	3 ± 44	7 ± 39	0.151

Data are presented as mean ± SD of patients.

A2C, apical two chamber; A4C, apical four chamber; IVMD, interventricular mechanical delay; LA, left atrium; LAX, long axis; LBBP, left bundle branch pacing; LV, left ventricle; LVEDV, left ventricular end-diastolic volume; LVEF, left ventricular ejection fraction; LVESV, left ventricular end-systolic volume; LVFW, left ventricle free wall; LV PEP, left ventricular pre-ejection period; N/A, not applicable; NS-HBP, non-selective His bundle pacing; PSD, peak systolic dispersion; RVFW, right ventricle free wall; RVP, right ventricular pacing; SPWMD, septal-to-posterior wall motion delay; TAPSE, tricuspid annular plane systolic excursion.

^†^ *P* < 0.05 when compared with control subjects.

^‡^ *P* < 0.05 when compared with RVP.

^§^ *P* < 0.05 when compared with LBBP.

^※^ *P* < 0.05 when compared with NS-HBP.

### 3.5 Left intraventricular mechanical synchrony

During sinus or intrinsic rhythm, parameters of LV mechanical synchrony, in terms of SPWMD, PSD, LV GLS, and Ts-LVFW-IVS, did not differ among the 4 groups at implant and at 6-month follow-up. However, during ventricular pacing, RVP group had longer SPWMD compared with LBBP and NS-HBP groups (122 ± 39 vs. 78 ± 22 vs. 82 ± 15 ms, respectively, *P* < 0.001) at implant and (113 ± 35 vs. 72 ± 23 vs. 86 ± 24 ms, respectively, *P* < 0.001) at 6-month follow-up, longer PSD compared with LBBP and NS-HBP groups (63 ± 17 vs. 53 ± 15 vs. 47 ± 20 ms, respectively, *P* = 0.027) at implant and (65 ± 26 vs. 48 ± 14 vs. 42 ± 22 ms, respectively, *P* = 0.002) at 6-month follow-up, lower LV GLS, especially over segments of anteroseptal and septal walls, compared with LBBP and NS-HBP groups (16.6 ± 5.4 vs. 20.0 ± 2.8 vs. 18.5 ± 5.8%, respectively, *P* = 0.033) at 6-month follow-up, and longer Ts-LVFW-IVS compared with LBBP and NS-HBP groups (83 ± 55 vs. 16 ± 37 vs. 29 ± 39 ms, *P* < 0.001) at implant and (45 ± 50 vs. 18 ± 38 vs. 15 ± 30 ms, respectively, *P* = 0.042) at 6-month follow-up (Table 2).

### 3.6 Right intraventricular mechanical synchrony

During sinus or intrinsic rhythm and ventricular pacing, the parameter of RV mechanical synchrony, in terms of Ts-RVFW-IVS, did not differ among the RVP, LBBP and NS-HBP groups at implant and at 6-month follow-up (Table 2).

### 3.7 Interventricular mechanical synchrony

During sinus or intrinsic rhythm, parameters of interventricular mechanical synchrony, in terms of LV PEP, IVMD and Ts-LVFW-RVFW, did not differ among the 4 groups at implant and at 6-month follow-up. However, during ventricular pacing, RVP group had longer LV PEP (141 ± 16 vs. 104 ± 23 ms, *P* = 0.014) compared with LBBP group at 6-month follow-up, longer IVMD compared with the LBBP group (14 ± 13 vs. −6 ± 6 ms, *P* = 0.032) at implant and (21 ± 24 vs. −8 ± 11 ms, *P* = 0.002) at 6-month follow-up, and longer Ts-LVFW-RVFW compared with the LBBP group (58 ± 60 vs. 1 ± 46 ms, *P* = 0.001) at implant (Table 2).

### 3.8 LV myocardial work analysis

**Figure 4:**
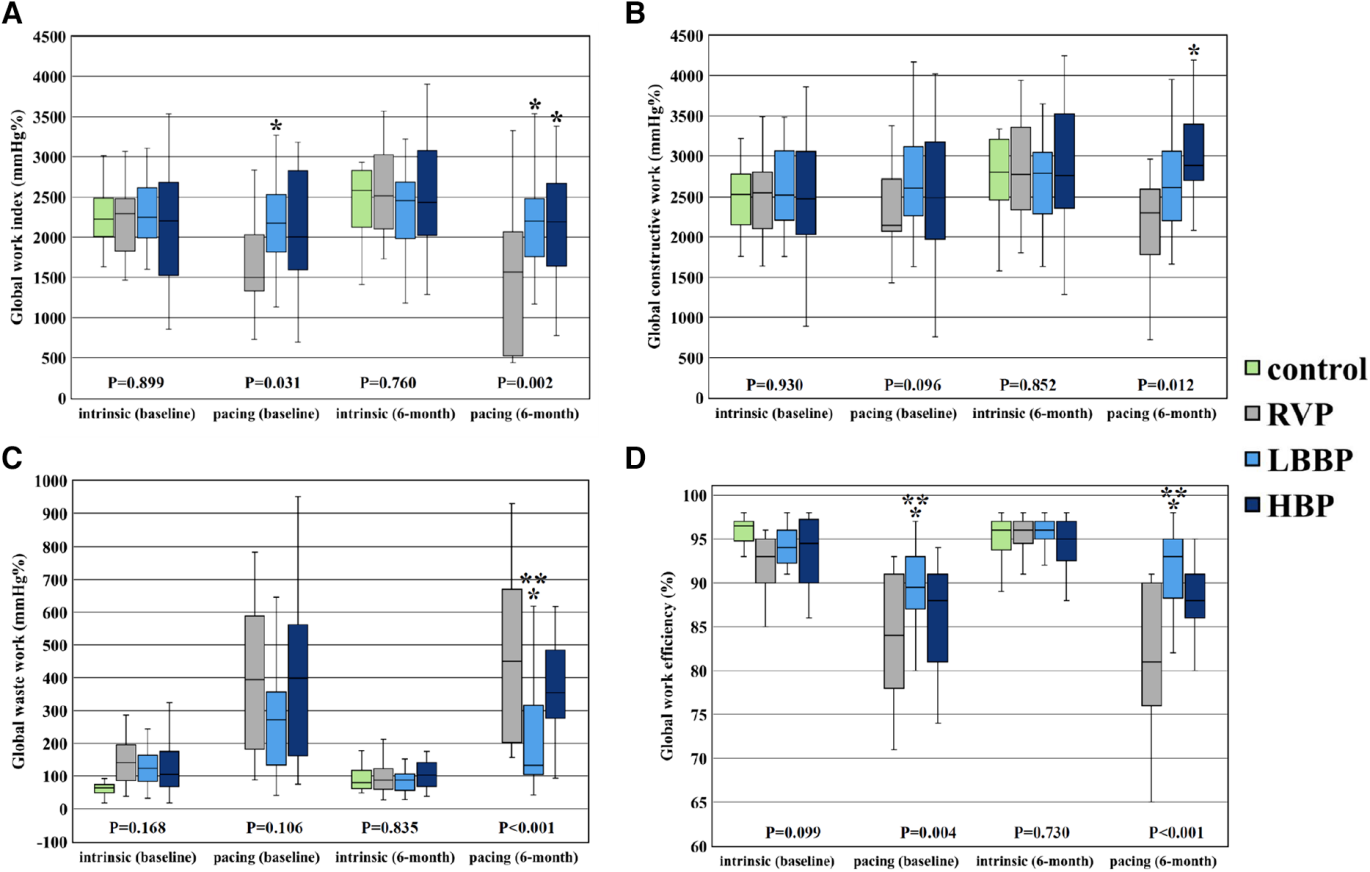
Parameters of global LV myocardial work. Comparison of global LV myocardial work parameters including global work index (**A**), global constructive work (**B**), global waste work (**C**), and global work efficiency (**D**) among RVP, LBBP and NS-HBP groups at implant, and 6-month follow-ups. Asterisk (*) means *P*-value <0.05 compared with RVP group; double asterisk (**) means *P*-value <0.05 compared with NS-HBP group. Abbreviations as in Figure 2.

There were no significant differences in all the parameters of MW (GWI, GCW, GWW and GWE) during sinus or intrinsic rhythm among the 4 groups at implant and at the 6-month follow-up (Table 3 and Figure 4). However, during ventricular pacing, LBBP group had higher GWI compared with RVP group (2,174 ± 529 vs. 1,689 ± 562 mmHg%, *P* = 0.023) at implant and (2,189 ± 527 vs. 1,493 ± 799 mmHg%, *P* = 0.002) at 6-month follow-up, and NS-HBP group had higher GWI compared to RVP group only at the 6-month follow-up (2,146 ± 703 vs. 1,493 ± 799 mmHg%, *P* = 0.010) (Table 3 and Figure 4). During ventricular pacing, NS-HBP group had higher GCW compared with RVP group (2,921 ± 771 vs. 2,203 ± 866 mmHg%, *P* = 0.009) at 6-month follow-up (Table 3 and Figure 4). During ventricular pacing, LBBP group had lower GWW compared with RVP and NS-HBP groups (211 ± 161 vs. 484 ± 281 vs. 406 ± 234 mmHg%, respectively, *P* < 0.001) at 6-month follow-up, and consequently, LBBP group had higher GWE compared with RVP and NS-HBP groups (89.5 ± 4.3 vs. 83.4 ± 7.2 vs. 85.0 ± 9.5%, respectively, *P* = 0.004) at implant and (91.4 ± 5.0 vs. 80.9 ± 8.3 vs. 86.4 ± 8.1%, respectively, *P* < 0.001) at 6-month follow-up (Table 3 and Figure 4).

**Table 3:** Myocardial work parameters during sinus or intrinsic rhythm and synchronized ventricular pacing rhythm in the control and different pacing groups at implant and 6-month follow-up.

	At implant					6-month follow-up				
	Control	RVP	LBBP	NS-HBP	*P*-value	Control	RVP	LBBP	NS-HBP	*P*-value
GWI (sinus or intrinsic rhythm), (mmHg%)	2,273 ± 378	2,222 ± 450	2,271 ± 402	2,165 ± 715	0.899	2,452 ± 471	2,552 ± 546	2,363 ± 480	2,495 ± 724	0.760
GWI (ventricular pacing), (mmHg%)	N/A	1,689 ± 562	2,174 ± 529^‡^	2,103 ± 733	0.031	N/A	1,493 ± 799^§^^※^	2,189 ± 527	2,146 ± 703	0.002
GCW (sinus or intrinsic rhythm), (mmHg%)	2,485 ± 413	2,495 ± 502	2,599 ± 496	2,537 ± 768	0.930	2,742 ± 527	2,846 ± 619	2,698 ± 498	2,837 ± 823	0.852
GCW (ventricular pacing), (mmHg%)	N/A	2,290 ± 525	2,755 ± 641	2,553 ± 906	0.096	N/A	2,203 ± 866^※^	2,645 ± 570	2,921 ± 771	0.012
GWW (sinus or intrinsic rhythm), (mmHg%)	65 ± 30	145 ± 74	157 ± 149	131 ± 91	0.168	91 ± 39	103 ± 74	92 ± 60	105 ± 44	0.835
GWW (ventricular pacing), (mmHg%)	N/A	412 ± 215	297 ± 197	394 ± 244	0.106	N/A	484 ± 281	211 ± 161^‡^^※^	406 ± 234	<0.001
GWE (sinus or intrinsic rhythm), (%)	96.0 ± 1.6	92.2 ± 3.4	93.5 ± 3.7	93.5 ± 4.0	0.099	95.3 ± 2.7	95.4 ± 2.6	95.4 ± 2.3	94.6 ± 2.9	0.730
GWE (ventricular pacing), (%)	N/A	83.4 ± 7.2	89.5 ± 4.3^‡^^※^	85.0 ± 9.5	0.004	N/A	80.9 ± 8.3	91.4 ± 5.0^‡^^※^	86.4 ± 8.1	<0.001

Data are presented as mean ± SD of patients.

GCW, global constructive work; GWE, global work efficiency; GWI, global work index; GWW, global wasted work; LBBP, left bundle branch pacing; N/A, not applicable; NS-HBP, non-selective His bundle pacing; RVP, right ventricular pacing.

^†^ *P* < 0.05 when compared with control subjects.

^‡^ *P* < 0.05 when compared with RVP.

^§^ *P* < 0.05 when compared with LBBP.

^※^ *P* < 0.05 when compared with NS-HBP.

## 4. Discussion

The main findings of this study were (1) LBBP group had less left intraventricular mechanical dyssynchrony during ventricular pacing compared with RVP group but had no difference in left intraventricular mechanical dyssynchrony compared to NS-HBP group at 6-month follow-up; (2) right intraventricular mechanical synchrony during ventricular pacing of LBBP group did not differ from that of NS-HBP and RVP groups; (3) LBBP group had less interventricular mechanical dyssynchrony during ventricular pacing compared with RVP group at 6-month follow-up; (4) LBBP group had better LV MW parameters (GWI, GWW and GWE) during ventricular pacing compared with RVP group and better LV MW parameters (GWW and GWE) during ventricular pacing compared with NS-HBP group at 6-month follow-up (Figure 4).

### 4.1 The left atrial size and LV systolic and diastolic function of LBBP

Long-term RVP can cause LV systolic dysfunction, pacing-induced cardiomyopathy and HF (1–4, 23). The mechanism of adverse effects is due to asynchronous electrical activation of LV caused by RVP-induced left bundle branch block pattern (3, 4, 23). Our recent study revealed that delta increment in pacing QRS duration, similar to electrical dyssynchrony, increased the risk of cardiovascular mortality in patients with pre-existing bundle branch block (24). Nahlawi et al. reported that RVP caused deterioration of LVEF after one-week continuous pacing, that persisted even after cessation of pacing 24 h later (25).

The left atrial size significantly increased during ventricular pacing at 6-month follow-up in the RVP group compared with the LBBP and NS-HBP groups (Table 2). Consequently, RVP increased the risks of atrial fibrillation, HF hospitalization and stroke (2).

### 4.2 The intraventricular and interventricular mechanical synchrony of LBBP

Interventricular and intraventricular mechanical dyssynchronies play a crucial role on ventricular pump function (3, 4, 17, 23). RVP can induce significant left intraventricular mechanical dyssynchrony, especially between the anteroseptal wall (with early activation) and the posterolateral wall (with late activation) (4, 9, 23). Recently, several studies reported that NS-HBP with high output (mean threshold 1.8 ± 1.2 V at 0.42 ms) and LBBP could prevent LV mechanical dyssynchrony, but NS-HBP with low output (mean threshold 1.0 ± 0.4 V at 0.42 ms) could not (9, 26). In this study, LBBP had better left intraventricular mechanical synchrony at implant and 6-month follow-up and better LV GLS, particularly over the anterior wall (LAX view) and septum (A4C view), at 6-month follow-up compared with RVP (Table 2). Moreover, LBBP provided stable threshold at long-term follow-ups compared with NS-HBP (Figure 3).

Interventricular dyssynchrony indicates a delay in activation between LV and RV, resulting in a lack of coordinated contraction between LV and RV. In HF patients, interventricular dyssynchrony was associated with higher risk of cardiac events, independent of the QRS duration and LVEF (18). In this study, LBBP had less interventricular mechanical dyssynchrony during ventricular pacing compared with RVP. An intra-patient-controlled study (10) reported that LBBP resulted in modest delay in RV activation and less interventricular synchrony measured by IVMD and Ts-LVFW-RVFW compared with NS-HBP in patients with bradycardia. However, in this study, LBBP, including 15 patients (32.6%) with pre-existing right bundle branch block, did not differ in interventricular synchrony compared to NS-HBP (Tables 1, 2).

### 4.3 LV myocardial work efficiency of LBBP

Global LV MW is a novel method to estimate LV systolic function, derived from computed LV pressure-strain loops incorporating both brachial cuff systolic blood pressure recordings and STE data (13, 14). Previous studies have demonstrated that there is a good correlation between LVEF and GWE in the general population, and GWE can be applied to assess the outcomes in patients with cardiac diseases (14, 15, 27, 28). Moreover, lower resting values of LV GLS and GWE in HF patients with preserved LVEF suggest an early subclinical myocardial damage, seemly associated with lower exercise capacity, greater pulmonary congestion, and blunted LV contractile reserve during effort (28). Furthermore, in the Geisinger-Rush Conduction System Pacing Registry, Sharma et al. (29) reported that LBBP patients with a ventricular pacing burden >20% had a lower risk of all-cause mortality, HF hospitalization, or upgrade to biventricular pacing compared to RVP. In an animal study, RVP caused significant redistribution of work away from the septum toward the LV lateral wall and significant redistribution of perfusion toward the LV anterolateral regions and a 30%–40% decreased myocardial efficiency (stroke work/myocardial oxygen consumption), whereas LV apical and septal pacing (via transventricular septal approach) did not significantly change myocardial efficiency (30). In this study, we first reported that during atrial synchronized ventricular pacing, the LBBP group had better GWE compared with RVP and NS-HBP groups (Table 3 and Figure 4). Further studies are warranted to assess that the better GWE achieved with LBBP compared with RVP in this study translates into better long-term clinical outcomes in different patient population with high burden ventricular pacing.

## 5. Limitation

There are several limitations in this study. First, this was a single-center prospective study with small sample size, and STE study only at implant and 6-month follow-up. Second, although several studies reported how to differentiate between LBBP and LVSP (20–22), it is still an inconclusive issue. In this study, we excluded 17 patients with LVSP using the algorithm reported by Chen et al. (20). Third, the echocardiographic images during non-selective LBBP with or without anodal capture were not distinguished in this study. Fourth, the worse GWW and GWE estimations during NS-HBP compared with LBBP might be attributed to partial right ventricular myocardial capture. Finally, individually regional LV myocardial work was not further evaluated during LBBP.

## 6. Conclusion

After 6-month of >40% ventricular pacing burden, LBBP resulted in less left intraventricular mechanical dyssynchrony and better LV MW parameters (GWI, GWW and GWE) compared with RVP, and had better MW parameters (GWW and GWE) compared with NS-HBP. Further studies are warranted to assess the association between GWE and long-term clinical outcomes in LBBP with high burden ventricular pacing.

## Data Availability

The original contributions presented in the study are included in the article/**Supplementary Material**, further inquiries can be directed to the corresponding author.
