# Supplementary material for: Left bundle branch pacing preserved left ventricular myocardial work in patients with bradycardia
Source: Front Cardiovasc Med. 2023 Sep 14;10:1201841. doi: 10.3389/fcvm.2023.1201841 (PMC10539618; doi:10.3389/fcvm.2023.1201841)
Supplement: Supplementary file 1 [file Datasheet1.docx]

***Supplementary Material***

**Left Bundle Branch Pacing Preserved Left Ventricular Myocardial Work in Patients with Bradycardia**

Huang-Chung Chen, MD, Wen-Hao Liu, MD, Yung-Lung Chen, MD, Wei-Chieh Lee, MD, Yen-Nan Fang, MD, Shaur-Zheng Chong, MD, Mien-Cheng Chen, MD

**Correspondence:** Mien-Cheng Chen: chenmien@ms76.hinet.net

**Supplemental methods: Procedures of conduction system pacing**

Twelve-lead electrocardiogram and intracardiac electrograms were simultaneously recorded using the multichannel Prucka CardioLab recording system (GE Medical Systems Information Technologies, Milwaukee, WI, USA).

For HBP, as previous description^1^, a 4.1-French lumenless lead (SelectSecure 3830, Medtronic Inc., Minneapolis, MN, USA) was delivered via a nondeflectable curve sheath (C315 His, Medtronic Inc., Minneapolis, MN, USA) and the lead tip was fixed into the His bundle area. Testing for pacing threshold was starting at 5.0 V @ 1 ms, and pacing threshold of either selective or nonselective HBP less than 2.0 V @ 1 ms was acceptable.^1^

For LBBP, the ventricular septal thickness was assessed by echocardiography before procedures. The delivery sheath (C315 His, Medtronic Inc., Minneapolis, MN, USA) was placed about 1 to 1.5 cm from His bundle site or septal leaflet of tricuspid vale toward RV apex (fluoroscopy right anterior oblique views 30°). A 4.1-French lumenless lead (SelectSure 3830, Medtronic Inc., Minneapolis, MN, USA) was advanced via the C315 His sheath and the lead tip was abutting the septum. Pacing at 5.0 V @ 0.4 ms was applied to create electrocardiographic QRS morphology of “W” pattern with the notch closer to nadir in lead V1, and then, the pacing lead was screwed perpendicularly into LV septum, and the advance was stopped till confirmation of capture of the LBB.^2^ According to previous report^2^, evidences for direct LBB capture were as follows: (1) pacing QRS morphology of a right bundle branch block (RBBB) pattern; (2) identification of the LBB potential; (3) pacing stimulus to left ventricular activation time (S-LVAT) shortens abruptly with increasing output or remains shortest and constant both at low and high outputs; or (4) selective LBBP and non-selective LBBP. Regarding to differentiate between LBBP and left ventricular septal pacing (LVSP), LBBP was considered (1) if LBB potential is recorded and S‐LVAT ≤85 ms, or (2) if LBB potential is not recorded but S‐LVAT ≤70 ms; LVSP was considered (1) if LBB potential is recorded but S‐LVAT >85 ms, or (2) if LBB potential is not recorded and S‐LVAT >70 ms.^3^

For RVP, the pacing lead (model 5076, Medtronic Inc., Minneapolis, MN, USA) was positioned in the RV septum.

**Reference**

1. Dandamudi G, Vijayaraman P. How to perform permanent His bundle pacing in routine clinical practice. *Heart Rhythm.* 2016;13:1362-1366.
2. Huang W, Chen X, Su L, Wu S, Xia X, Vijayaraman P. A beginner's guide to permanent left bundle branch pacing. *Heart Rhythm.* 2019;16:1791-1796.
3. Chen X, Qian Z, Zou F, et al. Differentiating left bundle branch pacing and left ventricular septal pacing: An algorithm based on intracardiac electrophysiology. *J Cardiovasc Electrophysiol.* 2022;33:448-45.
